# Macrophage migration inhibitory factor family proteins are multitasking cytokines in tissue injury

**DOI:** 10.1007/s00018-021-04038-8

**Published:** 2022-01-29

**Authors:** Shanshan Song, Zhangping Xiao, Frank J. Dekker, Gerrit J. Poelarends, Barbro N. Melgert

**Affiliations:** 1 Department of Molecular Pharmacology, Groningen Research Institute of Pharmacy, University of Groningen, Antonius Deusinglaan 1, 9713 AV Groningen, The Netherlands; 2 Department of Chemical and Pharmaceutical Biology, Groningen Research Institute of Pharmacy, University of Groningen, Antonius Deusinglaan 1, 9713 AV Groningen, The Netherlands; 3 University Medical Center Groningen, Groningen Research Institute of Asthma and COPD, University of Groningen, Hanzeplein 1, 9713 GZ Groningen, The Netherlands

**Keywords:** Receptors, Binding partners, Signal transduction, Divergent effects, Tissue repair

## Abstract

The family of macrophage migration inhibitory factor (MIF) proteins in humans consist of MIF, its functional homolog D-dopachrome tautomerase (D-DT, also known as MIF-2) and the relatively unknown protein named DDT-like (DDTL). MIF is a pleiotropic cytokine with multiple properties in tissue homeostasis and pathology. MIF was initially found to associate with inflammatory responses and therefore established a reputation as a pro-inflammatory cytokine. However, increasing evidence demonstrates that MIF influences many different intra- and extracellular molecular processes important for the maintenance of cellular homeostasis, such as promotion of cellular survival, antioxidant signaling, and wound repair. In contrast, studies on D-DT are scarce and on DDTL almost nonexistent and their functions remain to be further investigated as it is yet unclear how similar they are compared to MIF. Importantly, the many and sometimes opposing functions of MIF suggest that targeting MIF therapeutically should be considered carefully, taking into account timing and severity of tissue injury. In this review, we focus on the latest discoveries regarding the role of MIF family members in tissue injury, inflammation and repair, and highlight the possibilities of interventions with therapeutics targeting or mimicking MIF family proteins.

## Introduction

Macrophage migration inhibitory factor (MIF) is a ubiquitous protein with properties of a cytokine, a chaperone, and an enzyme [1–3]. MIF was initially discovered as a soluble factor from activated lymphocytes capable of inhibiting migration of macrophages during studies of delayed-type hypersensitivity [4, 5]. In follow-up research, MIF was recognized as a negative regulator of the immunosuppressive actions of glucocorticoids and since then MIF has been associated with inflammation as well. In 1989, MIF was heterologously expressed as a recombinant protein [6] and this enabled more elaborate investigations of the functions of MIF in various disease models. Importantly, production of recombinant MIF proteins also enabled resolution of its crystal structure [7]. In addition to its association with inflammation, a growing body of evidence demonstrates that MIF influences a variety of molecular processes important for the maintenance of cellular homeostasis including promotion of cellular survival, anti-oxidant signaling, angiogenesis, and tissue repair [8–16].

Tissue repair is a complex and dynamic interplay between various cell types which are intricately regulated by a dense signaling network of cytokines, growth factors or hormones. The process of tissue repair can be divided into four phases: hemostasis, inflammation, repair, and resolution. Interestingly, MIF was found to be involved throughout these four dynamic and overlapping tissue repair stages. Upon injury, immediate repair of damaged blood vessels is needed to prevent extensive blood loss and this importantly involves coagulation. In this phase, MIF has been found to a play key role in maintenance of hemostasis through promoting platelet survival and attenuating vascular leakage [10, 17]. The second stage of repair is characterized by inflammatory responses to prevent micro-organisms from entering the wound and to start up tissue repair. In this phase MIF attracts immune cells and is associated with the release of other inflammatory factors, such as IL-1β and TNFα [18, 19]. In the repair and resolution phases of tissue repair, MIF also acts as a growth factor to promote survival and proliferation of endothelial cells, fibroblasts and epithelial cells [20–22]. Therefore, many divergent functions of MIF have been observed during tissue repair, which are mediated through different receptors or binding partners.

In addition to MIF influencing tissue repair, the other MIF family members, D-dopachrome tautomerase (D-DT, also known as MIF-2) and DDT-like protein (DDTL), may also be involved. The overall structure of D-DT is highly similar to that of MIF, and D-DT has been suggested to have similar biological functions to those of MIF [23]. DDTL, the third and most recently reported member of the family, shows high sequence identity to D-DT, but little is known about the functional properties of the DDTL protein [24].

In this review, we will discuss intracellular and extracellular activities of MIF and D-DT to understand their multiple functions in nonpathological and pathological processes connected to tissue repair. In addition, we also outline new concepts that have been introduced in therapeutics for diseases associated with MIF and/or D-DT.

## MIF and its family members

**Fig. 1:**
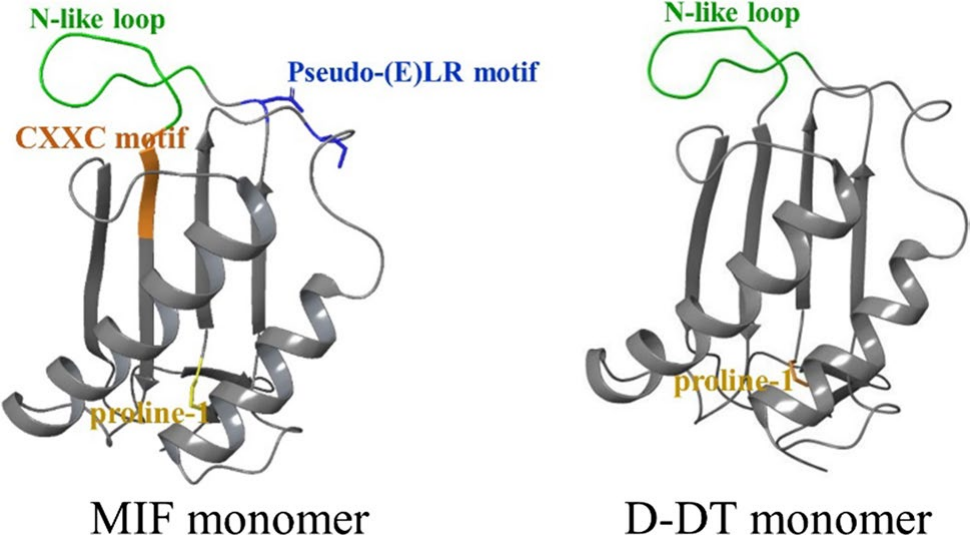
The MIF and D-DT monomers. The proline-1 residue (yellow), the N-like loop (green), the CXXC motif (orange), and the pseudo-(E)LR motif (blue) are highlighted and identified. All structures used were obtained from the Protein Data Bank (PDB, http://www.rcsb.org/pdb/home/home.do) and superimposed with PyMOL

The MIF gene is highly conserved and has been found in mammals, fish, nematodes, protozoa, and even in bacteria and plants [25, 26]. The sequence identity between these MIF protein orthologs from different species ranges from 20 to 100%. Human MIF has 90% sequence identity with mouse and rat MIF [27]. The MIF gene in the human genome is located on chromosome 22 (22q11.2) and two polymorphisms of this gene have been implicated in human disease. One is a single-nucleotide G to C mutation at position − 173 in the 5' flanking region and the other is a CATT-tetranucleotide repeat at position − 794 [28]. Enzymatically active MIF is a homotrimer with each monomer being a peptide of 114 amino acids that folds into four β-strands and two α-helices [29]. The characteristic N-terminal proline residue (proline-1) is essential for the enzymatic activity of MIF, which catalyzes the keto-enol tautomerization of substrates such as D-dopachrome and 4-hydroxylphenylpyruvate (4-HPP). MIF reportedly also has thiol-protein oxidoreductase activity, which is dependent on a Cys–Ala–Leu–Cys (CXXC) motif at positions 56–59, with Cys-59 being essential for activity [30]. D-DT on the other hand, lacks Cys-59 and therefore this thiol-protein oxidoreductase activity (Fig. 1).

MIF is constitutively expressed in most tissues [31] and stored in intracellular vesicles and in the nucleus [32, 33]. As MIF lacks an N-terminal signal sequence for translocation into the endoplasmic reticulum (ER)/Golgi, it has been suggested that MIF secretion occurs through a noncanonical pathway, but the actual mechanism of MIF secretion is still unknown [34, 35]. In addition, MIF has also been found to be secreted in exosomes. Exosome-derived MIF enhanced heart function, inhibited reactive oxygen species generation and apoptosis, and promoted metastasis [36]. A recent study by Dankers et al. added important information to our knowledge of the secretion mechanism, demonstrating that MIF release is induced by necrosis, receptor-interacting kinase-3-dependent programmed necrosis (also known as necroptosis), and NLRP3 inflammasome-dependent pyroptosis [37]. Extracellular MIF can enter into the cell cytosol again through endocytosis mediated by its membrane receptors [22]. Therefore, MIF exerts its biological functions through these membrane receptors but also through multiple intracellular partners.

The second member of the MIF family is encoded by the D-dopachrome tautomerase (D-DT) gene [23]. D-DT was first reported in 1993 and was characterized as a tautomerase in human melanoma and liver [38]. The similarities and differences between MIF and D-DT have been discussed before in an elegant review by Merk and colleagues [39]. In humans, the D-DT gene is located close to the MIF gene (~ 80 kb apart) on chromosome 22 (22q11.2). D-DT has an overall structure that is similar to that of MIF, but their amino acid sequence similarity is limited (34% pairwise sequence identity in humans and 27% in mice). D-DT has not been studied in great detail yet and relatively little is known about its functions in comparison to MIF.

DDTL has approximately 70% sequence identity with D-DT and its encoding gene is located in close proximity to the genes coding for D-DT and MIF on chromosome 22. To date, it is not clear if the gene is expressed to yield DDTL protein in humans and the biological function of this putative protein is also unknown. Our recent study showed that DDTL mRNA is produced in human lung tissue and that mRNA levels are not different between control lung tissue and lung tissue of patients with chronic obstructive pulmonary disease (COPD) [24].

## Membrane receptors

**Fig. 2:**
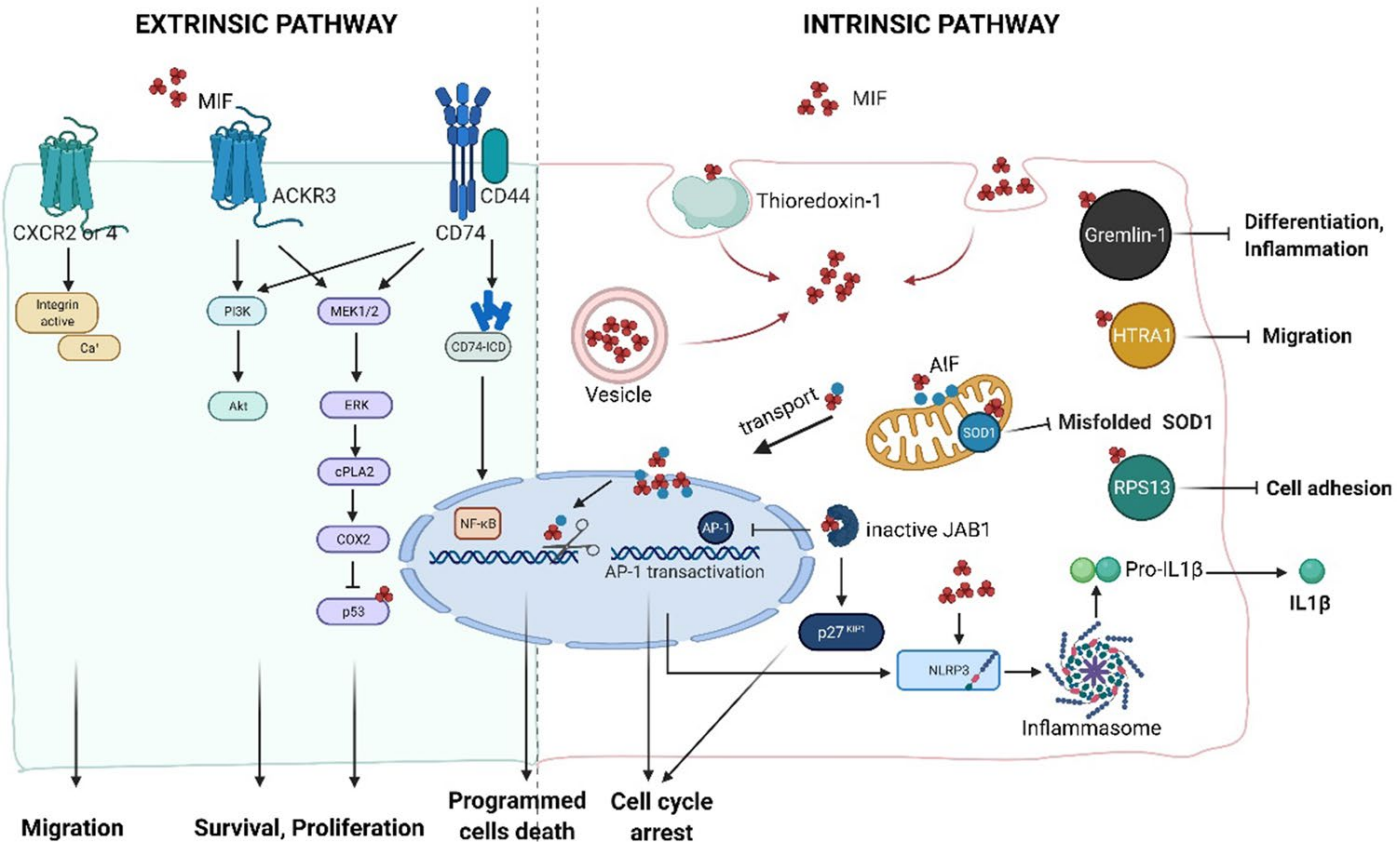
MIF-induced signaling pathways (Created with BioRender.com). MIF mediates its biological activities either via membrane receptors (left panel) or through intracellular binding partners (right panel). *Extrinsic pathway* MIF binds to its membrane receptor CD74 or/and atypical chemokine receptor 3 (ACKR3), which initiates a phosphorylation signal through activation of the mitogen-activated protein kinase (MAPK) and the phosphoinositide 3-kinase (PI3K) pathway. The downstream effects of MIF increase cellular survival and proliferation through the inhibition of p53. The intracellular domain (ICD) of CD74 could enter into nucleus and signal through nuclear factor-κB (NF-κB). The interaction of MIF-CXCR2/4 induces cell migration. *Intrinsic pathway* Depending on the oxidative environment, cytosolic MIF interacts with thioredoxin-1, resulting in internalization of MIF. Intracellular MIF binds to superoxide dismutase 1 (SOD1) and inhibits the accumulation of misfolded SOD1, protecting from motoneuron damage. Under ischemic or excitotoxic stress, apoptosis-inducing factor (AIF) binds to MIF and guides MIF into the nucleus. Nuclear MIF works as a nuclease and causes DNA damage, leading to cell death. Intracellular MIF interacts with C-Jun activation domain-binding protein-1 (JAB1), inhibiting JAB1-induced transcription of AP-1 and degradation of cyclin-dependent kinase inhibitor 1B (p27Kip1), resulting in cell cycle arrest. Upon damage or infection, intracellular MIF interacts with nitrogen permease regulator-like 3 (NLRP3) and facilitates the interaction between NLRP3 and vimentin, resulting in IL1β release. MIF has been identified as the first endogenous inhibitor of HTRA1, which prevents the inhibition of astrocyte migration. Gremlin-1 also binds to MIF with high affinity, which results in MIF-dependent inflammation and cell differentiation

MIF has been reported to be involved in inflammation and cell proliferation through four membrane receptors: CD74 and chemokine receptors CXCR2, CXCR4, and CXCR7 (also known as ACKR3). Furthermore, the epidermal growth factor receptor (EGFR) has recently been identified as a novel receptor for O-GlcNAcylated MIF, but further studies are needed to confirm the interaction and induced-downstream signaling. In contrast, CD74 was the only membrane receptor described for D-DT but we have recently shown that D-DT can also interact with ACKR3 and stimulate lung epithelial repair [23] (Fig. 2).

### CD74

The most studied membrane receptor for MIF is CD74, also known as HLA class II histocompatibility antigen gamma chain. In line with its role as a MHC class II chaperone, the highest CD74 expression is observed on the surface of antigen-presenting cells like dendritic cells, B cells, and macrophages [40]. Upon MIF engagement, CD74 recruits CD44 and subsequently mediates downstream signal transduction, through pathways such as PI3K/Akt and ERK1/2 [41]. However, in chronic lymphocytic B cells, CD74 was also identified as a transcription regulator. Binding of MIF to CD74 resulted in the cleavage of CD74 by SPPL2A protease to release a CD74 intracellular domain (CD74-ICD) [42]. CD74-ICD then interacted with p65 to enter into the nucleus and modulated transcription of NF-κB target genes and thereby rescuing cells from apoptosis and promoting cell proliferation [43]. To date, the precise binding site of MIF to CD74 is not clear, but small molecules and mutations targeting the tautomerase active site pocket have been shown to interfere with the binding of MIF to CD74 [44]. Interestingly, Fan et al. produced a single cysteine mutant of MIF (MIF^N110C^) that covalently locks MIF into a trimer and demonstrated that the trimeric form of MIF was able to bind to CD74 but lost the ability to induce downstream signaling, suggesting that the oligomerization of MIF monomers is essential for MIF-CD74 induced signal transduction [45]. In a biological context MIF can be produced as monomers, dimers, and/or trimers [46]. Therefore, knowing the concentrations of different MIF oligomeric forms and understanding their ability to assemble the active CD74 receptor complex may be crucial in studying MIF/CD74-mediated functions.

D-DT has also been shown to bind to CD74, albeit with an approximately three-fold higher binding rate and an 11-fold faster dissociation rate compared to MIF [23]. This finding suggests there is difference between the biological effects of MIF and D-DT. An example of this is the finding that D-DT binding to CD74 not only triggers a signaling cascade but also leads to D-DT internalization [23].

### CXCR2/4

MIF has also been shown to directly bind to chemokine receptors CXCR2 and CXCR4, which regulate inflammation and cell migration via the PI3K/Akt and ERK1/2 pathways [47]. Moreover, CXCR2 and CXCR4 are also able to form receptor complexes with CD74 and can be internalized together with CD74 after interaction with MIF, which also results in activation of the PI3K/Akt and ERK1/2 pathways [47–49]. Theoretically, most chemokines bind to their receptors via a two-site binding involving the N-like loop and Glu-Leu-Arg (ELR) motif. MIF has an ELR-like motif consisting of Asp-44 and Arg-11 that was found to bind to exoloop regions of CXCR2. MIF also has a N-like loop consisting of residues 46–55 that was found to bind the N-domain of CXCR2 [50, 51]. The N-like loop is also involved in the interaction between MIF and CXCR4 and therefore the binding site for CXCR4 slightly overlaps with that of CXCR2 [52–54]. In addition, tautomerase inhibitor ISO-1 and mutations to the catalytic proline-1 residue inhibit the interaction of MIF with CXCR4, suggesting that the proline-1 residue is also required for optimal MIF-CXCR4 interaction [55].

D-DT lacks the ELR motif which is necessary for interaction with CXCR2 [23, 39]. Therefore, D-DT is not able to bind to CXCR2 and is less involved in recruiting monocytes and leukocytes than MIF [23]. To date, the interaction between D-DT and CXCR4 is still not clear, and our work has shown no evidence of it being able to bind [56].

### ACKR3 (CXCR7)

In addition to CXCR2 and CXCR4, MIF also is a ligand for atypical chemokine receptor 3 (ACKR3, formerly known as CXCR7) [10, 21, 57]. MIF was shown to bind to the N-terminal sequence stretch of ACKR3 and then initiate downstream signaling pathways [57]. For instance, MIF was found to activate the PI3K/Akt pathway via ACKR3, independent of CXCR4 and CD74 in platelets and T cells [10]. However, MIF was not able to activate the ERK1/2 pathway through ACKR3 when CD74 and CXCR4 were not present. In cells that express CD74 and/or CXCR4, such as monocytes and B cells, ACKR3 forms heteromeric receptor complexes with CD74 and/or CXCR4 resulting in MIF-mediated activation of EKR1/2 [21]. These findings suggest that ACKR3 could modulate MIF signaling as part of a CD74/CXCR4/ACKR3 complex or serve as a separate receptor for MIF. For D-DT again the evidence is scarce and only our own studies looked into interactions between D-DT and ACKR3. In these studies, we found that D-DT contributed to lung alveolar epithelial repair via interactions with ACKR3 [56].

### Epidermal growth factor receptor (EGFR)

EGFR is a tyrosine kinase receptor and plays an essential role in proliferation, survival and differentiation of epithelial cells. Zhang et al*.* found that naturally secreted O-GlcNAcylated MIF competitively binds to EGFR and blocks epidermal growth factor (EGF)-induced activation of EGFR, ERK1/2 and c-Jun signaling, cell invasion, and brain tumor formation [58]. O-GlcNAcylation of MIF at Ser112 and Thr113 is required for MIF to bind to EGFR, therefore only MIF purified from human cells is able to bind to EGFR, but not MIF heterologously expressed in bacterial systems. However, no further study has been reported until now, and the interaction between EGFR and MIF needs to be further investigated and clarified.

## Intracellular partners

Apart from acting as a cytokine through its membrane receptors, MIF can also exert effects in the intracellular space directly. MIF is stored in vesicles in the cytosol and also can be endocytosed through a clathrin-dependent mechanism [59]. In the intracellular space, several proteins have been reported to bind to MIF which resulted in different types of effects such as cell survival, programmed cell death, and inflammation among others (Fig. 2). In contrast, little information is available on intracellular partners that mediate D-DT actions.

### Apoptosis-inducing factor (AIF)

One of the newly described binding partners of MIF is apoptosis-inducing factor (AIF) that plays a role in a process called parthanatos. This is a caspase-independent cell-death pathway that is distinct from apoptosis, necrosis, or other known forms of cell death [60]. Well-known diseases involving parthanatos include neurological disorders like Parkinson's disease, stroke, and multiple sclerosis [13]. Parthanatos depends on excessive activation of the central DNA damage sensor protein poly(ADP-ribose) polymerase 1 (PARP1) and is mechanistically dependent on nuclear AIF translocation. During certain types of cellular stress overactivation of PARP-1 triggers release of AIF from mitochondria. MIF was found to interact with AIF and be recruited by AIF to the nucleus where MIF cleaves DNA into fragments leading to cell death [60, 61]. Three-dimensional modeling of MIF revealed that structure of trimeric MIF shows domains that are characteristic for the PD-D/E(X)K super-family of nucleases and therefore has nuclease activity. Monomers do not have PD-D/E(X)K topology and therefore monomeric MIF does not have nuclease activity. MIF has both 3′ exonuclease and endonuclease activity which allows MIF to digest genomic DNA into large fragments. The nuclease activity is independent from the oxidoreductase and tautomerase activity of MIF. Inhibition of MIF's nuclease activity by mutation of its nuclease domain or by disruption of its protein–protein interaction with AIF markedly attenuated ischemic neuronal cell death and acute brain injury in mice. This interaction between MIF and AIF was confirmed by another study which showed MIF knockdown protected neurons from oxidative stress-induced parthanatos associated with spinal cord injury [62]. In this regard, it would be interesting to determine whether cytosolic MIF contributes to DNA damage in other types of cell injuries too. Interactions of D-DT with AIF have not been reported yet.

### p53

In 1999, Hudson et al*.* demonstrated that MIF can interact with p53 and inhibit p53 activity [63]. p53, a tumor suppressor protein, is expressed at low or even undetectable levels in homeostatic tissue, while in response to cellular stress (DNA damage, oncogene activation and hypoxia) intracellular p53 increases significantly and plays important roles in cell cycle arrest, apoptosis, and senescence [64]. This ensures that damaged or abnormal cells are not able to proliferate, which is crucial in tissue damage to prevent tumor growth [65]. MIF was found to inhibit this p53 activity, therefore allowing proliferation of cells and possibly tumor development. Further studies indicated that MIF can directly bind to p53 in mammalian cells [64, 66]. This interaction was significantly reduced by a mutation in the cysteine-81 residue of MIF, suggesting that Cys81 is essential for association between MIF and p53. Endogenous expression of MIF in different cell types lowered p53 levels and suppressed p53 nuclear localization, thereby preventing its transcriptional activity resulting in inhibition of p53-dependent senescence and apoptosis [67, 68]. Genetic deletion of MIF resulted in G0/G1 cell cycle arrest and suppression of proliferation in fibroblasts by the p53-dependent pathway [69]. Both endogenously expressed and exogenously added MIF were able to inhibit apoptosis by overcoming p53-mediated growth arrest or apoptosis [64, 67, 70].

Another recent study demonstrated that MIF and D-DT cooperatively inhibit steady state p53 phosphorylation, stabilization and transcriptional activity in human lung adenocarcinoma cell lines. The combined loss of MIF and D-DT by siRNA led to dramatically reduced cell cycle progression, clone formation and increased programmed cell death when compared to loss of either MIF or D-DT alone [71].

### Superoxide dismutase (SOD1)

A recent discovery highlighted a novel role for intracellular MIF in regulating the accumulation of misfolded Cu/Zn superoxide dismutase (SOD1). Mutations in SOD1 are associated with 20% of the cases of familial amyotrophic lateral sclerosis, which is characterized by loss of motor neurons [72]. Translocation and accumulation of misfolded SOD1 in mitochondria and/or endoplasmic reticulum has been identified as a cause of motor neuron death. However, the expression of SOD1 is ubiquitous. Why is accumulation of misfolded SOD1 then selective to nervous system tissues? Based on this question, Israelson et al*.* did a study on nonnervous system tissue and demonstrated that MIF in cytosolic extracts from liver cells was a key factor inhibiting accumulation of SOD1 in mitochondrial membranes [73]. This observation was further verified in neuronal cells showing that recombinant MIF inhibited misfolded mutant SOD1 binding to mitochondrial and endoplasmic reticulum membranes [73]. Furthermore, studies in mice showed that deletion of endogenous MIF accelerated disease onset and progression, and shortened survival of mutant SOD1 mice [12, 74]. Another study from Israelson’s group, using real time surface plasmon resonance, showed that MIF could directly interact with SOD1 [75]. However, the binding site is still unclear. It has been suggested that switching from multimeric to monomeric forms of MIF, exposes a hydrophobic surface that can provide chaperone activity for misfolded mutant SOD1 [73]. The interaction between MIF and misfolded SOD1 is again independent of its tautomerase and oxidoreductase activity as similar chaperone activity was found using MIF mutants lacking tautomerase or oxidoreductase activity [75]. In addition, MIF^N110C^, a cysteine mutant of MIF and unable to induce CD74-dependent signaling, showed potent inhibition of misfolded SOD1 and higher affinity for SOD1 compared to wildtype MIF [75]. This finding suggests that the chaperone activity of MIF is independent of its CD74-mediated cytokine activity. Interactions of D-DT with SOD1 have not been studied yet.

### Jun-c activation domain-binding protein (JAB1) and hepatopoietin (HOP)

JAB1 has been reported to promote cell proliferation by acting as a co-activator of the transcription factor activator protein 1 (AP-1) and by degradation of the cyclin-dependent kinase inhibitor p27^Kip1^ [22, 76]. MIF can interact with JAB1 and can prevent JAB1-induced transcription of AP-1 pathways and degradation of p27^Kip1^ resulting in cell cycle arrest [77]. Both endogenously expressed and exogenously added MIF can interact with JAB1. Interestingly, one study found that the binding ability of MIF to JAB1 was reduced and the activity of AP-1 was increased in HepG2 cells which were co-transfected with HOP and MIF. HOP is a liver specific-regeneration stimulator, which upregulates the AP-1 pathway through interaction with JAB1 [78]. Both HOP and MIF have conserved (Cys–Ala–Leu–Cys) motifs which are important for oxidoreductase activity. Usually, proteins with these motifs form heterodimers through disulfide linkages [53]. In yeast cells, MIF was found to bind to HOP with higher affinity than HOP to JAB1 [78]. Therefore, the dynamic balance of MIF/HOP/JAB1 complex formation in liver tissue may be critical for liver regeneration.

### Ribosomal protein S19 (RPS19)

Ribosomal proteins are a family of RNA-binding proteins that are essential for the translation of messenger RNA into protein. Ribosomal protein S19 (RPS19) is one of 80 types of ribosomal proteins and known as a component of the 40 S small subunit of the ribosome and therefore an integral part of the protein translation machinery [79]. However, RPS19 also exists in a free form in the cytosol and can be released from cells to have extracellular functions [80]. Filip et al. found that RPS19 interacted directly with MIF resulting in inhibition of monocyte adherence to endothelial cells in vitro by blocking the binding between MIF and CD74 or CXCR2 respectively [80]. In 2013, Lan and colleagues first showed the effects of the MIF-RPS19 interaction in vivo [81]. They demonstrated that RPS19 treatment suppressed expression of MIF and CD74 in a mouse model of anti-glomerular basement membrane glomerulonephritis and downregulated the MIF-CD74 induced activation of the ERK1/2 pathway. This then resulted in significantly attenuated development of glomerular crescents and glomerular necrosis, and prevented renal dysfunction and proteinuria [81]. A few years later, they further confirmed the protective effects of RPS19 treatment in a mouse model of cisplatin-induced acute kidney injury, showing downregulation of MIF/CD74-induced inflammation, which was similar to results found with MIF knock-out mice [19].

### Gremlin-1

Gremlin-1 (also known as Drm) belongs to the DAN/Cerberus protein family, which is part of the cysteine knot superfamily that includes TGFβ and VEGF. Gremlin-1 is a bone morphogenetic protein (BMP) antagonist and it binds to BMP2, 4, and 7. Gremlin-1 has also been shown to bind to MIF with high affinity which resulted in lowering MIF-induced release of TNFα from macrophages [82]. In ApoE^−/−^ mice, that spontaneously develop atherosclerotic lesions, treatment with Gremlin-1 fused to an Fc tail of an antibody (Gremlin1-Fc), resulted in fewer macrophages in atherosclerotic lesions and attenuated atheroprogression compared to treatment with inactivated Gremlin1-Fc. Although not conclusively proven, these data suggest that Gremlin-1/MIF interaction is critically involved in plaque biology and progression.

The interactions between Gremlin-1 and MIF may also be important in regulation of monocyte function and survival in atherosclerosis. Gremlin-1 was found to inhibit MIF-dependent monocyte migration and adhesion to activated endothelial cells in vitro and to injured carotid arteries in mice in vivo [83]. Furthermore, Gremlin-1 also inhibited MIF-induced differentiation of monocytes into macrophages in these same studies. The Gremlin-1 C-terminal region was found to be critical for regulating MIF-modulated chemotactic activity because the Gremlin-1 mutant R172A, with an exchange of arginine to alanine is this region, failed to inhibit MIF-induced monocyte differentiation into macrophages [84]. These data indicate that Gremlin-1 is an endogenous antagonist of MIF and also suggest a role for Gremlin-1/MIF interaction in atherosclerosis.

### High temperature requirement A1 (HTRA1)

HTRA1 is a serine protease which belongs to the HTRA family which is highly conserved from prokaryotes to humans [85]. The majority of HTRA1 is secreted into the extracellular space and then degrades extracellular matrix proteins such as fibronectin, type II collagen, elastin, and bone sialoprotein and cleaves growth factors like fibroblast growth factor 8 [86, 87]. HTRA1 also acts intracellularly, binding transforming growth factor β (TGFβ) family proteins such as BMP4, GDF5, and TGFβ itself, to downregulate cellular signaling [87]. MIF was recently identified as the first endogenous inhibitor of HTRA1 [88, 89]. In mouse primary astrocytes, MIF was shown to be co-expressed with HTRA1 and prevented the inhibition of astrocyte migration by inhibiting HTRA1. The first 38 amino acids of MIF, which contain the first α-helix, were found to be important for the interaction with HTRA1, but the exact interaction site with HTRA1 is not clear. Molecular modeling indicated that the PDZ domain of HTRA1 may interact with the loop between the N-terminal β-sheet and the first α-helix of MIF, while the protease domain of HTRA1 interacts with the first α-helix [89].

### Thioredoxin-1 and thioredoxin-interacting protein

Thioredoxin-1 is a ubiquitous redox protein and protects proteins from oxidative stress damage and can regulate programmed cell death [90]. Bernhagen and colleagues first demonstrated that MIF possess a CXXC (Cys–Ala–Leu–Cys) motif with Cys-56 and Cys-59 being critical for thiol-protein oxidoreductase activity [91]. Son and colleagues found that MIF formed a complex with thioredoxin-1 in ATL-2 cells and also in culture supernatant of ATL-2 cells [92]. An anti-thioredoxin-1 antibody inhibited MIF endocytosis, and MIF internalization was also not observed in Jurkat T cells that do not express thioredoxin-1 on the cell surface. These data suggest that cell surface thioredoxin-1 can play an important role in MIF internalization into cells [92].

Thioredoxin-interacting protein, also known as vitamin D3 up-regulated protein-1 or thioredoxin-binding protein-2, is a known inhibitor of NF-κB activity [93]. Interestingly, MIF was also able to directly interact with thioredoxin-interacting protein via Cys^57^ and Cys^81^ of MIF and Cys^36^ and Cys^120^ of thioredoxin-interacting protein. Data from Kim et al*.* suggest that MIF induces NF-κB activity by counteracting the inhibitory effect of thioredoxin-interacting protein on the NF-κB pathway via direct interaction with thioredoxin-interacting protein [94]. Taken together, these findings suggest that under conditions of oxidative stress, the interactions between MIF/thioredoxin-1/thioredoxin-interacting protein modulate inflammatory responses.

### NLR family pyrin domain containing 3 (NLRP3)

Several studies have indicated links between MIF and the IL-1 family proteins such as IL-1β and IL-18. Both in vivo and in vitro studies have shown that MIF-deficient mice/cells or mice/cells treated with MIF inhibitors secrete less IL-1β and IL-18 in response to a variety of inflammatory stimuli compared to controls [95, 96]. IL-1β and IL-18 secretion are dependent on activation of inflammasomes, which are cytosolic multiprotein oligomers that after activation and assembly promote proteolytic cleavage, maturation and secretion of IL-1β and IL-18. A recent study has shown a specific role for MIF in activation of inflammasome-dependent IL-1β and IL-18 release through interaction with NLPR3 [18]. This is one of the essential components of multiprotein complex that embodies the inflammasome. MIF was found to be required for the interaction between NLRP3 and the intermediate filament protein vimentin, which is critical for NLRP3 activation and subsequent IL-1β and IL-18 release.

### Insulin

Insulin is an essential hormone in the coordination of systemic glucose homeostasis and is secreted by pancreatic β-cells. MIF was found to be secreted together with insulin by pancreatic β-cells and to act as an autocrine factor to stimulate insulin release [97]. An anti-MIF antibody and the absence of MIF inhibited glucose-stimulated insulin release resulting in the development of obesity, glucose intolerance and hyperglycemia [98, 99]. Furthermore, MIF also acts as a chaperon involved in insulin biosynthesis. Insulin from MIF-deficient mice was poorly functional and completely unable to trigger glucose uptake into the hepatocytes [100]. In addition, insulin from MIF-deficient mice had a different conformation or posttranslational modification compared to insulin from wildtype mice. MIF actually stimulated insulin hexamer formation in cell-free systems [100]. Thus, it seems that MIF can act as an adaptor protein for insulin oligomerization. Insulin was also found to co-immunoprecipitate with insulin in lysates from pancreatic islets, which further suggests that MIF has a role in protein folding of insulin and reinforces the chaperone role of MIF [98, 101].

With respect to D-DT and insulin interactions, a direct relationship between D-DT and insulin is still unknown, but D-DT levels in adipose tissue in insulin-resistant mice were lower than in the control mice. In addition, treatment of this insulin resistance with recombinant D-DT improved glucose intolerance caused by obesity, suggesting that the observed low levels of D-DT in these mice are in part responsible for impaired glucose-stimulated insulin secretion [102].

## The expressions of MIF family proteins

**Fig. 3:**
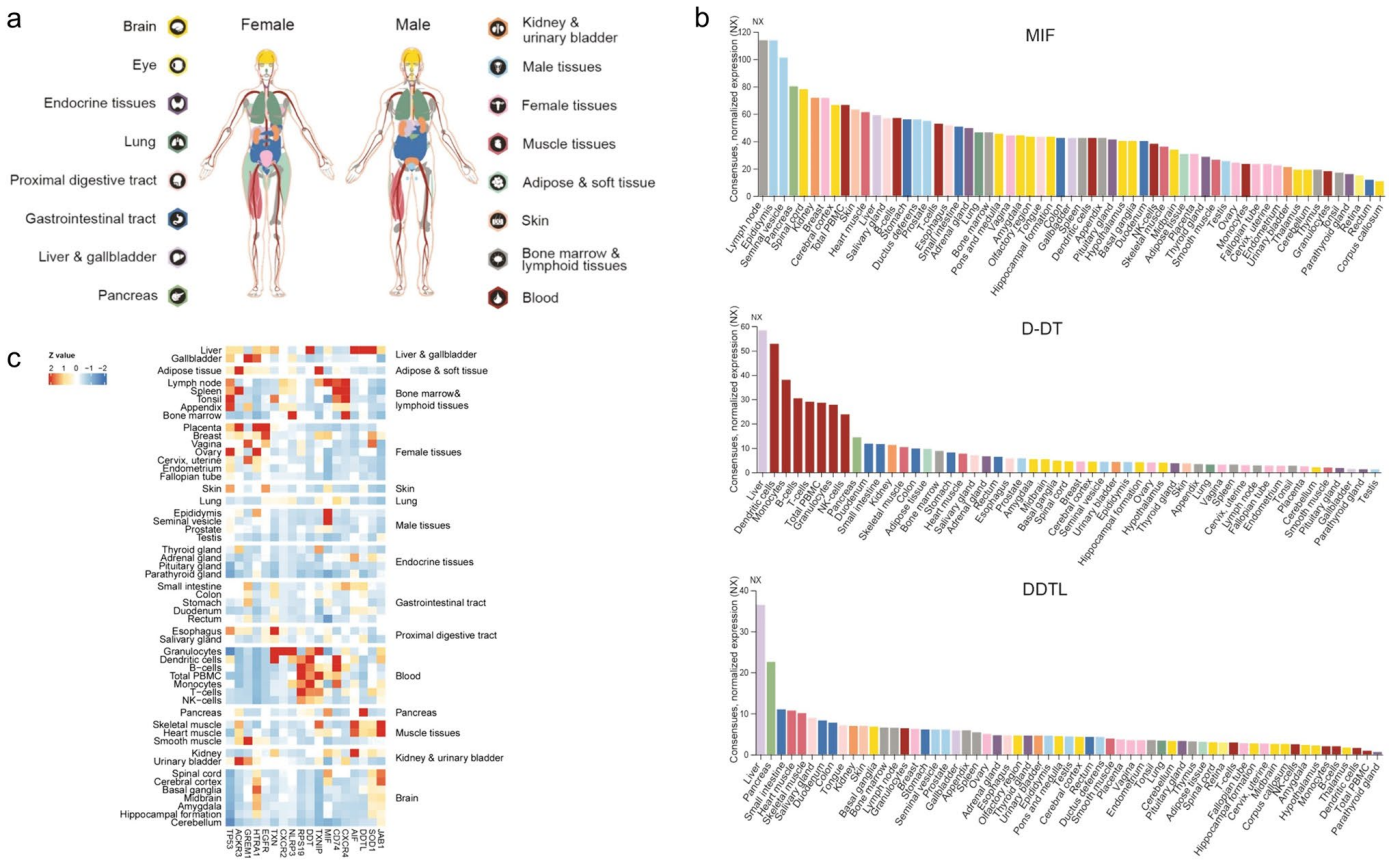
Expression of MIF family proteins in human tissues [103–105]. **a** Overview of the transcriptome of tissues and organs analyzed by the three independent consortia Human Protein Atlas (HPA), FANTOM5, and GTEx. In total, 16 organ systems (with several tissues comprising an organ system) were used to create a consensus normalized expression based on the expression levels of all three datasets. **b** A summary of the normalized MIF, D-DT and DDTL gene expression in human tissues in 61 different tissues and cells. The colors are corresponding to the color of each organ in (**a**). **c** Heatmap of normalized expression z-values computed for MIF family genes in different tissues

To understand MIF family proteins in human physiology and disease, we mapped expression levels of MIF family proteins and their partners across different organs, tissues, and cell types and studied expression levels in a tissue-restricted manner. According to the open-access database www.proteinatlas.org [103–105], MIF is expressed in all tissues of the human body, corresponding to 16 different organ systems, including blood. In addition, both D-DT and DDTL are highly expressed in liver, while D-DT is also greatly produced by white blood cells (Fig. 3a-b).

To further investigate the expression of their binding partners, we used transcriptome data from www.proteinatlas.org [103–105]. Figure 3 shows that MIF and its partners, CXCR4, CD74 NLRP3, ACKR3, and p53, are highly expressed in bone marrow and lymphoid tissues. However, in both female and male reproductive tissues, the expression of MIF is higher compare to D-DT and DDTL. This suggests that MIF maybe more important than D-DT and DDTL in reproductive system diseases. In contrast, white blood cells greatly express D-DT but not MIF nor DDTL, which suggests that D-DT may play the leading role in innate inflammation through CD74. In brain tissue, MIF and its intracellular partners, JAB1, SOD1, and HTRA1 are expressed in high levels (Fig. 3c).

## Tissue injury

The underlying biological effects of MIF, D-DT and their receptors and related partners in tissue injury have been investigated in many organs. Here, we have summarized the roles of MIF and D-DT in kidney, liver, brain and lung injury because these have been the most studied types of injuries with the most solid evidence for MIF (Table 1). However, D-DT has not been studied much in any type of injury and therefore information about its role is still limited.

**Table 1:** In vivo evidence for therapeutic targeting of MIF in tissue injury

Tissue	Models	Treatments	Regulated cell types	Cellular source of MIF	Results	References
Kidney	Mice: unilateral ischemia/reperfusion or rhabdomyolysis induced acute kidney injury	MIF knock out	More apoptotic and necroptotic tubular cells, more infiltration of inflammatory cells such as Erhr3 + macrophages, F4/80 + macrophages, dendritic cells, and Ly6G + granulocyte	Unknown	MIF-deficient mice had worse acute tubular injury than wild-type mice	[9, 110]
	Mice: cisplatin or ischemia/reperfusion-induced acute kidney injury	MIF knock out, ribosomal protein S19	Less tubular necrosis, less infiltration of F4/80 + macrophages, CD3 + T cells, and neutrophils	Tubular epithelial cells	MIF-deficient mice had less kidney injury; inhibiting MIF with ribosomal protein S19 could reduce kidney injury	[8, 19]
Liver	Mice: chronical ethanol-induced liver injury	MIF knock out and a MIF inhibitor	More apoptosis of hepatic macrophages	Hepatocytes	MIF-deficient mice had less liver injury compared to wild-type mice	[112, 114]
	Mice: acute ethanol-induced liver injury	MIF knock out and a MIF inhibitor	Do not affect activation of neutrophils and macrophages	Hepatocytes	MIF deletion and MIF inhibition prevented mice from injury	[116]
	Mice concanavalin A-induced liver injury	MIF knock out	Less hepatocyte necrosis and recruitment of activated T cells	Unknown	Deletion of MIF protected mice from liver injury compared to wild-type mice	[115]
	Mice: CCl4 and thioacetamide-induced liver fibrosis	MIF knock out and rmMIF treatment	Suppressed activation of hepatic stellate cells	Unknown	MIF-deficient mice had more fibrosis than wild-type mice	[118]
Brain	Mice: Middle cerebral artery ligation	MIF knock out and rmMIF treatment	More apoptotic neurons	Unknown	MIF-knockout mice had greater infarct size than wild type mice. rmMIF treatment rescued neurons from oxidative stress	[124]
	Mice: Transient middle cerebral artery occlusion	MIF knock out or MIF inhibitor ISO-1	Less neuronal death and more microglia	Cortical parvalbumin-positive interneurons	MIF deficiency or administration of MIF antagonist ISO-1 resulted in a smaller infarct size	[17, 125, 126]
Lung	Mice: cigarette smoke-induced COPD	MIF knock out	More pulmonary apoptosis of endothelial and alveolar epithelial cells	Unknown	MIF-deficient develop aged-related emphysema, exposure to cigarette smoke aggravated this emphysema	[11, 20]
	Mice: ozone-induced COPD	MIF inhibitor ISO-1	Fewer macrophages	Unknown	MIF inhibitor ISO-1 improved lung function in mice exposed to ozone	[128]
	Rats: lipopolysaccharide-induced acute lung injury	MIF antibody	Less LPS-induced neutrophil accumulation in the lungs	Alveolar macrophages and bronchial epithelial cells	MIF antibody protects against lipopolysaccharide-induced acute lung injury	[131]

### Kidney injury

Acute kidney injury is a global public health problem with high morbidity and mortality and is defined as a rapid (within hours) and reversible decline in kidney function. Until now, dialysis is the only reliable therapeutic intervention [106]. To understand the underlying pathophysiology of acute kidney injury, animal models of ischemia/reperfusion injury or drugs/toxins injury (cisplatin, aristolochic acid, folic acid, etc.) present similar pathological features to the human disease and are therefore the most commonly used. Tubular epithelial cells are sensitive to many types of injury and are the key players in the pathological progression of acute kidney injury [107]. Incidentally, these cells are also the main producers of MIF in acute kidney injury [19].

In a cohort of patients experiencing renal ischemia after cardiac surgery, circulating MIF levels increased greatly [9], suggesting an association between renal injury and MIF release. Furthermore, elevated plasma and urinary MIF levels were found in patients with acute kidney injury compared to healthy controls. When the kidney injury resolved, MIF levels dramatically decreased again [8]. The upregulation of MIF also correlated positively with inflammatory mediators like IL1β and kidney injury molecules like creatinine [8, 108]. This suggests that serum levels of MIF are related to the severity and progression of kidney injury. However, in patients with SLE urinary MIF was also increased, but was not linked to active renal disease [109], therefore the role of MIF is unclear.

Data from animal models help a little to generate clarity. In a murine model of ischemia/reperfusion injury, MIF-deficient mice have been shown to have significantly worse acute tubular injury than wild-type mice [110]. An elegant study from Stoppe et al*.* identified MIF as the protective factor in both ischemia/reperfusion and rhabdomyolysis-induced acute kidney injury [9]. Compared to wild-type mice, deletion of MIF significantly aggravated acute kidney injury as shown by higher serum creatinine levels. MIF-deficient mice also had more apoptotic tubular cells, increased infiltration of inflammatory cells, and a higher tubular injury score compared to wild type mice [9]. Recombinant MIF treatment was able to mitigate this acute kidney injury through inhibiting cell death of tubular cells [9]. Therefore, from these studies MIF appears to have a protective role and may be upregulated after injury to mitigate the injury.

However, in contrast to the above-mentioned studies, a study by Li and colleagues demonstrated that MIF contributed to ischemia/reperfusion-induced acute kidney injury in mice [8]. MIF-deficient mice had significantly less tubular necrosis, fewer infiltration of inflammatory cells and lower expression of pro-inflammatory cytokines. These results were replicated in a model of cisplatin-induced acute kidney injury and further supported by data showing that inhibiting MIF with ribosomal protein S19 could reduce kidney injury. Using a mutant version of this inhibitory ribosomal protein S19 that was not able to inhibit MIF had no effects on the induced acute kidney injury [19]. These data suggested that MIF also has a harmful role in kidney injury. The reason for these dual effects of MIF in acute kidney injury remains unknown. Of note, three different murine models of acute kidney injury were used in the Stoppe study and all three models suggested that MIF is protective when there is minimal renal injury. However, when renal injury is more severe like in models of cisplatin or ischemia/reperfusion-induced injury, MIF appears to promote progression of acute kidney injury. MIF levels have been shown to correlate with disease severity and therefore the release of MIF in more severe acute kidney injury maybe significantly higher than in mild acute kidney injury. The higher levels of released MIF may then induce more widespread events associated with inflammation (like infiltration of immune cells) that may contribute to progression of injury, while lower levels only stimulate local repair mechanisms. Therefore, MIF may have opposite effects based on the time and severity of acute kidney injury.

As mentioned before little is known about the role of D-DT in injury. One study investigated the role of D-DT in a murine model of ischemia/reperfusion-induced kidney injury using D-DT knockout mice. These mice had more severe acute tubular injury than wild type mice and similar injury to MIF knockout mice [110]. Interestingly, treatment of MIF knockout mice with D-DT significantly ameliorated tubular injury suggesting that MIF and D-DT may have similar roles in ischemia/reperfusion-induced acute kidney injury.

### Liver injury

The liver is an extremely important organ for metabolism and detoxification of harmful compounds. These compounds come from ingested foods, intestinal bacteria, as well as ingested environmental toxins. Thus, the liver is exposed to potentially harmful compounds on a daily basis but fortunately has a large capacity for regeneration. Liver disease can develop after massive or prolonged exposure and is characterized by hepatocellular damage, inflammatory cell infiltrating in the hepatic parenchyma, and tissue remodeling, ultimately resulting in progressive fibrosis and cirrhosis [111].

Studies in patients with liver disease, showed that circulating MIF in serum was significantly higher in patients with alcohol-related liver disease than in healthy individuals and, importantly, positively correlated with disease severity [112, 113]. Barnes et al*.* showed that MIF-deficient mice chronically exposed to ethanol had less liver injury compared to wild-type mice, suggesting a harmful role for MIF during liver injury [114]. Importantly, MIF appears to be produced by nonmyeloid cells, particularly hepatocytes, in response to chronic ethanol feeding. Marian et al*.* used chimeric mice with deletion of MIF in myeloid or nonmyeloid cells specifically and found that only deletion of MIF in nonmyeloid cells protected mice from chronic ethanol-induced liver injury [112]. Similarly, in a model of concanavalin A-induced T cell-mediated liver injury, deletion of MIF protected mice from liver injury compared to wild-type mice by inhibiting hepatocyte necrosis and recruitment of inflammatory cells [115]. In contrast, mice with MIF deficiency or treated with a MIF inhibitor were found to have aggravated liver injury shortly after ethanol-induced injury, but less injury in the long run and this was shown to be mediated through an effect on the unfolded protein response [116]. The unfolded protein response is a cellular stress response to unfolded or misfolded proteins in the lumen of the endoplasmic reticulum. In the acute phase, the unfolded protein response preserves cell function and is intended for cell survival, but prolonged disruption will steer the cell towards apoptosis [117]. MIF was shown to protect against acute ethanol-induced liver injury by preventing the unfolded protein response, but in the long run this led to more liver injury and an exacerbated unfolded protein response [116]. These data indicated that the role of MIF in liver injury depends on the stage and severity of the injury and suggests caution when thinking of MIF-directed therapies in liver injury.

In more severe models of liver damage, like toxin-induced liver fibrosis, Heinrichs et al*.* showed that MIF played an anti-fibrotic role in CCl4 and thioacetamide-induced liver fibrosis [118]. MIF-deficient mice had significantly more fibrosis with higher α-SMA expression and collagen deposition, but MIF deficiency had no influence on inflammatory cell recruitment. Moreover, systemic treatment with recombinant MIF ameliorated CCl4-induced fibrogenesis in mice. Importantly, this study also showed that this MIF treatment directly suppressed activation of hepatic stellate cells, the main producers of scar tissue in liver fibrosis, through a CD74-driven pathway. MIF was found to promote the phosphorylation of adenosine monophosphate-activated protein kinase in a CD74-dependent manner which inhibited hepatic stellate cell activation by platelet-derived growth factor.

In summary, the role of MIF in liver injury is not fully understood with studies showing conflicting results. However, while some studies clearly show that MIF contributes to acute liver injury with effects on inflammation and hepatocyte apoptosis in ethanol-induced models of liver injury, other studies support a hepato-protective role of MIF in liver fibrosis. A weakness of current murine ethanol-feeding models is that mice do not develop significant hepatic fibrosis. Therefore, a protective role of MIF during fibrosis-development due to excessive exposure to toxins may therefore not be visible. Thus, MIF seems to have divergent effects during liver injury promoting acute injury, but protecting against fibrosis development.

To date, only one study has looked into effects of D-DT on liver injury [119]. This study found dramatically higher levels of D-DT in livers of mice exposed to CCl4, suggesting D-DT may also have an important role in toxin-induced liver fibrosis in addition to MIF.

### Brain injury (cerebral ischemia)

Cerebral ischemia is an important cause of death and disability worldwide and is most often caused by blocking of blood vessels due to thrombosis resulting in brain damage [120]. The period of ischemia and the subsequent reperfusion injury cause many changes to the brain tissue involving importantly neurons, microglia and endothelial cells. Most of the brain damage is caused by insufficient blood supply and lack of nutrients, protein aggregation, oxidative stress, and glutamate excitotoxicity. The result of these processes is apoptotic or necrotic cell death which can lead to irreversible brain damage [121].

As MIF was shown to have mitigating effects on oxidative stress [122, 123], it has also been shown to have protective effects in cerebral ischemia. MIF was shown to rescue neurons from oxidative stress induced apoptosis by inhibiting caspase-3 activation, and MIF-knockout mice also had more dead neurons, as well as a greater infarct size after induction of an experimental stroke [124].

In contrast, in a model of transient middle cerebral artery occlusion, MIF was found to contribute to stroke pathology in mice and neuronal death in vitro, with knockout of MIF or administration of MIF antagonist ISO-1 resulting in a smaller infarct size [17, 125, 126]. In addition, MIF was recently found to act as a nuclease and thereby contributes to ischemic neuronal cell death [13, 62]. Together, these data suggest a detrimental role for MIF in brain ischemia.

To sum up, the role of MIF during brain ischemia was found to be either protective by suppressing neuron apoptosis or detrimental by promoting neuron death through interactions with different proteins. Like with other organs, these conflicting results illustrate our incomplete understanding of MIF and leave a lot of room for further research.

The role of D-DT in brain injury has not been investigated yet except for the fact that D-DT was shown to be widely expressed in the adult mouse brain and robustly expressed in heterogeneous interneurons, suggesting a function for D-DT in the brain as well [127].

### Lung injury

The lung can be affected by many types of pathologies related to injury and the most common type of chronic injury-related disease is chronic obstructive pulmonary disease (COPD) [128]. It is the fourth leading cause of death globally and is characterized by loss of alveolar tissue called emphysema and/or chronic inflammation of the airways called chronic bronchitis [129]. The most common cause in the Western world is exposure to cigarette smoke, but also exposure to indoor or outdoor air pollution are important other causes. The exposure to these noxious gases cause lung injury associated with an increase in inflammation, oxidative stress, cellular senescence and apoptosis [128].

In the lung too, the evidence for MIF being protective or harmful is conflicting. Higher levels of MIF protein and mRNA were shown in serum, sputum, lung tissue and in macrophages present in bronchoalveolar lavage of COPD patients compared to healthy smokers and non-smoker controls [24, 128, 130]. However, other studies showed lower levels of MIF in serum of patients with severe COPD compared to controls [20], and also lower plasma MIF levels in COPD patients compared to healthy smokers [11]. These divergent results were reproduced in an animal model for COPD. Mice exposed to cigarette smoke for three months had higher MIF levels in bronchoalveolar lavage fluid whereas mice exposed for six months had lower MIF levels compared to nonexposed controls and this coincided with emphysema development [11]. In addition, both MIF-deficient and CD74-deficient mice were found to develop aged-related emphysema and MIF-deficient mice developed even worse emphysema when exposed to cigarette smoke than MIF-deficient mice exposed to air [11, 20]. These data suggest that MIF is involved in protecting the alveoli during aging, and insufficient levels in COPD may contribute to emphysema development. However, MIF was also shown to aggravate COPD-like disease in animal models. MIF inhibitor ISO-1 improved lung function in mice exposed to ozone by inhibiting infiltration of immune cells into the lung [128]. Moreover, a MIF antibody significantly inhibited recruitment of neutrophils into the lungs of rats after being exposed to lipopolysaccharide, a model of acute lung injury [131]. The emerging pattern from these data is that again MIF appears to be harmful in cases of acute injury, but is more beneficial during repair of long-term injury. During acute injury MIF seems to promote harmful inflammation, whereas in tissue repair it may be protective through inhibition of epithelial apoptosis.

The data for D-DT and lung injury/COPD are again scarce. We recently showed that D-DT mRNA levels were higher in lung tissue of patients with COPD compared to controls, but these did not correlate with any measure of lung function investigated in this study [24]. In a recently published abstract by the American Thoracic Society, the authors investigated D-DT in the context of cigarette smoking and found that D-DT deficient mice were more susceptible to both spontaneous and cigarette smoke-induced emphysema compared to wild-type mice, similar to what was found for MIF-deficient mice [132]. In addition, upon exposure to acute hypoxia, D-DT-deficient mice succumb to lethal oxidative stress faster than wild type control mice, suggesting a role for D-DT in countering oxidative stress too [132]. Our own studies into the role of D-DT in lung tissue showed that D-DT treatment contributes to proliferation and differentiation of primary lung epithelial progenitor cells and D-DT may therefore be important in lung repair [56].

## MIF-targeting therapeutics

From the previous parts, it is obvious that MIF, and probably D-DT too, is involved in tissue injury and repair and possesses versatile functions. Currently, there are several therapeutic strategies including small molecule-, antibody- and peptide-based approaches that could be employed as possible therapies for tissue injury.

### Antagonists of MIF

As we discussed above, MIF-deficient mice show less damage during liver and brain injury. Therefore, blocking MIF activity may be a potential strategy to attenuate inflammation-induced injury. Several therapeutic modalities have been applied to inhibit MIF-related activities, such as MIF-neutralizing antibodies, MIF-mimic antagonist peptides, and MIF-directed small-molecule inhibitors [133]. Among these potential therapeutics, small-molecule inhibitors of MIF are among the most studied strategies. These inhibitors can be divided into two groups. One group consists of inhibitors blocking the protein–protein interactions between MIF and its receptors directly, such as MN-166 [134]. The other group includes the majority of reported MIF inhibitors and interfere with MIF-related activity through binding to the tautomerase-active site. Although the physiological function of MIF tautomerase enzyme activity is still elusive, some small-molecule inhibitors targeting this site were found to be effective in interfering with MIF-receptor interactions and therefore inhibited MIF-induced biological signaling. ISO-1 is one of the most used and studied small molecules targeting MIF and was found to have anti-inflammatory properties attenuating acute kidney and lung injury in many models [29, 135]. In addition, many research groups have discovered several more potent tautomerase inhibitors such as Jorgensen-3b, NVS 2, and Dekker 7, that exhibit nanomolar-level binding affinity for MIF [136–138].

To block protein–protein interactions, using neutralizing antibodies is currently a prevalent strategy. The application of MIF antibodies provided benefits in several diseases. Monoclonal antibody NIH/IIID.9 is one of most widely used MIF antibodies, which improved diseases such as atherosclerosis in pre-clinical models. The oxidized-MIF recognizing antibody imalumab has advanced into phase 1/2a clinical trials (NCT01765790) for colorectal cancer and lupus nephritis [139, 140].

As an alternative to small-molecule inhibitors and antibodies, several MIF-specific peptide-based therapies are now being investigated preclinically [133]. An octapeptide containing residues 79–86 of MIF was capable of competing for the binding with CD74, indicating its potential to inhibit MIF binding to CD74 [141]. Several other fragments of MIF such as MIF 47–56 and MIF 50–65 are also antagonists of MIF [51] and significantly blocked MIF/CXCR2 binding. A note of caution is required here though, because MIF and D-DT are important for growth of many different progenitor cells [132, 142, 143]. Even though MIF has shown protective effects on inflammation-induced injury, antagonism of MIF actions may not be ideal when damaged tissue needs progenitor cells to restore tissue function**.**

### Agonist of MIF

Considering the beneficial effects of MIF and D-DT during injury, MIF and D-DT may offer interesting therapeutic opportunities for tissue injury via boosting the endogenous regenerative ability of organs. Therefore, MIF agonists have also gained attention. Wang et al*.* reported a MIF agonist, MIF20, which can limit cardiac ischemic injury in mice through augmenting adenosine monophosphate-activated protein kinase phosphorylation and stimulating subsequent cellular glucose uptake [144]. In an earlier study, MIF20 was found to be a MIF agonist that can enhance the binding of MIF to CD74 in an ELISA assay [145]. Furthermore, Qi et al*.* have shown that treatment with D-DT protected isolated hearts against injury and contractile dysfunction after ischemia–reperfusion. The protective effect of D-DT also required activation of adenosine monophosphate-activated protein kinase, which was also mediated through a CD74-dependent mechanism [146]. However, again a word of caution is needed, because MIF (and maybe D-DT) are also important in promoting tumor growth and nurturing a local tumor microenvironment through chronic inflammation [66, 147–149]. Patients with chronic tissue injury already have an increased risk of developing cancer [150] and thereby treatment of tissue injury with MIF or D-DT may be a double-edged sword.

## Conclusion

MIF, and probably also D-DT, is a multifaceted protein interacting with multiple-binding partners and participating in many processes during tissue injury. However, the literature is confusing or even contradictory with respect to the functions of MIF in tissue injury. The divergent effects are explained by the cellular context, timing, and diverse physiological conditions, as well as the known and unknown pathways in which MIF is involved. For instance, as a pro-inflammatory cytokine MIF increased IL1β production which can impair tissue regeneration [151]. As a nuclease, MIF induced cell death. Furthermore, MIF interacts with JAB1 resulting in cell cycle arrest. As a growth factor, however, MIF can promote proliferation of tissue progenitor cells by interacting with CD74 or/and ACKR3 or inhibiting HTAR1/p53. Therefore, any treatment involving MIF (or D-DT) modulation needs to be carefully considered in context of the disease that is treated. Unwanted side effects are likely due to the many opposing functions described for MIF. A deeper understanding of MIF functions therefore seems warranted before long-term MIF-related treatments are tried out clinically.

In cancer, MIF clearly seems to be pathogenic and targeted treatment seems to be less controversial. Overexpression of MIF has been identified in many different types of cancers such as cancer of the colon, lung, breast, prostate, bladder, and in glioblastoma, cervical adenocarcinoma, and melanoma [148, 154–158]. In addition, knockout and inhibitors of MIF suppressed cancer cell growth. This evidence suggests that MIF is a promising target for cancer treatment. D-DT was also found to be essential for development of pancreatic ductal adenocarcinoma, cervical cancer, melanoma, liver cancer, lung cancer and renal cancer [148, 159–163]. In the latter, D-DT was found to drive renal tumorigenesis more than MIF, since D-DT knockdown in mice inhibited growth more than knock down of MIF [163]. Furthermore, several reports have demonstrated that MIF cooperates with D-DT in pancreatic ductal adenocarcinoma, cervical cancer, and non-small cell lung carcinoma [159, 160, 162]. Therefore, dual inhibitors of MIF and D-DT may be needed for the treatment of cancer.

In contrast to cancer, the different expression patterns of MIF and D-DT also support different roles of MIF and D-DT in noncancerous tissues. In healthy tissues, lymph nodes and male tissues such as male epididymis and seminal vesicle express the highest levels of MIF. D-DT, on the other hand, is most dominantly expressed in liver and immune cells such as dendritic cells, monocytes, T cells, B cells and NK cells. Upon injury or after specific stimulations, production patterns of MIF are also different from D-DT. For instance, in critically ill patients serum level of both MIF and D-DT were elevated compared to control, but the levels of D-DT were most profoundly upregulated [152]. In contrast, in patients with systemic sclerosis, MIF serum levels were significantly higher than in healthy controls while levels of D-DT are comparable to healthy controls [153]. Finally, after macrophages were stimulated with LPS, both D-DT and MIF production were increased in a similar pattern. However, production of MIF was around 20 times higher than production of D-DT [23]. Taken together the data suggest that D-DT is produced by other cells than MIF and that its production is also distinct from MIF during injury, indicating D-DT may not be a backup for MIF but also has its own distinct functions.

However, in many studies it remains uncertain whether the described pathological/physiological processes are truly MIF-mediated or whether its homologue D-DT (or their combination) is in fact responsible for some of them. For example, D-DT is promising therapeutic target candidate in heart failure and the role of MIF is only small [164]. Future therapeutic, diagnostic and prognostic use of MIF should therefore also take into consideration the contribution of D-DT and maybe also DDTL. Since far fewer binding partners have been described for D-DT, this protein may have better therapeutic options than MIF itself. However, this could also be the result of fewer studies into D-DT and this needs to be established in more detail first.

## Data Availability

All data and materials generated or analysed for this study are included in this published article.
